# Burden of Household Environmental Tobacco Smoke on Medical Expenditure for Japanese Women: A Population-Based Cohort Study

**DOI:** 10.2188/jea.JE20120072

**Published:** 2013-01-05

**Authors:** Toshitaka Morishima, Yuichi Imanaka, Tetsuya Otsubo, Kenshi Hayashida, Takashi Watanabe, Ichiro Tsuji

**Affiliations:** 1 Department of Healthcare Economics and Quality Management, Kyoto University Graduate School of Medicine, Kyoto, Japan; 1 京都大学大学院医学研究科医療経済学分野; 2 Department of Medical Informatics and Management, University Hospital of Occupational and Environmental Health, Kitakyushu, Fukuoka, Japan; 2 産業医科大学病院 医療情報部; 3 Division of Epidemiology, Department of Public Health and Forensic Medicine, Tohoku University School of Medicine, Sendai, Japan; 3 東北大学大学院医学系研究科社会医学講座公衆衛生学分野

**Keywords:** secondhand smoke, tobacco smoke pollution, longitudinal study, environment and public health, health care costs

## Abstract

**Background:**

The economic consequences of environmental tobacco smoke (ETS) have been simulated using models. We examined the individual-level association between ETS exposure and medical costs among Japanese nonsmoking women.

**Methods:**

This population-based cohort study enrolled women aged 40 to 79 years living in a rural community. ETS exposure in homes at baseline was assessed with a self-administered questionnaire. We then collected health insurance claims data on direct medical expenditures from 1995 through 2007. Using generalized linear models with interaction between ETS exposure level and age stratum, average total monthly expenditure (inpatient plus outpatient care) per capita for nonsmoking women highly exposed and moderately exposed to ETS were compared with expenditures for unexposed women. We performed separate analyses for survivors and nonsurvivors.

**Results:**

We analyzed data from 4870 women. After adjustment for potential confounding factors, survivors aged 70 to 79 who were highly exposed to ETS incurred higher expenditures than those who were not exposed. We found no significant difference in expenditures between moderately exposed and unexposed women. Total expenditures were not significantly associated with ETS exposure among survivors aged 40 to 69 or nonsurvivors of any age stratum.

**Conclusions:**

We calculated individual-level excess medical expenditures attributable to household exposure to ETS among surviving older women. The findings provide direct evidence of the economic burden of ETS, which is helpful for policymakers who seek to achieve the economically attractive goal of eliminating ETS.

## Abstract (ja)

**【背景】:**

受動喫煙は数多くの喫煙関連疾患の罹患リスクを上昇させることが知られている。受動喫煙と医療費の関係を検証した先行研究はいずれも計量モデルによって推計したものであり、実証研究は国内外において報告されていない。本研究の目的は、能動喫煙をしたことのない成人女性を対象に、家庭での受動喫煙が医療費を増やすかどうかを明らかにすることである。

**【方法】:**

宮城県大崎保健所管内に居住する国民健康保険加入者を追跡した大崎コホート国保研究のデータを用いた。1994年に40～79歳の加入者に受動喫煙に関する情報を質問紙で調査した。加入者の医療費データを保険者を通して1995年～2007年に収集した。一般化線形モデルを用いて様々な交絡因子を調整した上で、生存者/非生存者別に受動喫煙の高度暴露群、中等度暴露群、非暴露群の医療費を解析した。

**【結果】:**

4870人の成人女性の医療費を分析した。70～79歳の女性のうち、高度暴露群の生存者の月間総医療費（入院医療費＋入院外医療費＋調剤医療費）は非暴露群の生存者の医療費に比べて有意に高かった。中等度暴露群と非暴露群の間に有意差はなかった。他の年齢階級の生存者と全年齢階級の非生存者の医療費で受動喫煙の暴露レベルによる有意差を認めなかった。

**【結論】:**

受動喫煙によって医療費が増加することを個人レベルの観察データに基づいて実証した。受動喫煙によってもたらされる経済的負担を示した本研究は、受動喫煙を防止する政策を形成するための１つの客観的根拠となるだろう。

## INTRODUCTION

Exposure to environmental tobacco smoke (ETS), also known as secondhand smoke, passive smoking, and involuntary smoking, is a risk factor for mortality^1^^,^^2^ and morbidity from many diseases, including lung cancer^3^^,^^4^ and coronary heart disease.^5^^,^^6^ ETS exposure accounts for 11% of all tobacco-related deaths.^7^ Öberg et al^8^ reported that 1.0% of worldwide mortality is attributable to ETS. Public health concern is therefore elevated because diseases attributable to ETS are occurring among nonsmokers, and the resulting increase in mortality and morbidity is involuntary.

Medical expenditures are higher for active smokers than for nonsmokers,^9^^–^^15^ and ETS is a risk factor for many of the same diseases caused by active smoking.^3^^–^^6^ These facts naturally lead us to question whether ETS increases medical expenditures. Little is known, however, of the relationship between ETS exposure and medical expenditures among adults. Previous studies estimated the economic burden attributable to ETS by combining ETS-attributable incidence of diseases and medical costs associated with those diseases.^16^^–^^21^ The results were obtained from simulations using economic models and did not necessarily reflect the real world.

Japanese women have the highest ETS exposure among the 7 major industrialized nations.^22^ The World Health Organization (WHO) has calculated that 49% to 62% of Japanese women are exposed to ETS.^22^ WHO also reported that 42% of Japanese men smoked in 2006, which was the highest rate among the 7 nations. However, the prevalence of smoking among Japanese women was 13% in 2006—the lowest among the 7 nations.

We used individual-level observations of a single cohort of Japanese women to examine differences in direct medical expenditure among nonsmokers and those highly exposed, moderately exposed, and not exposed to ETS.

## METHODS

### Study design and setting

We used a prospective cohort design. The data were derived from the Ohsaki Cohort Study, the details of which have been reported elsewhere.^23^ In brief, this study started in 1994. We conducted a questionnaire survey of National Health Insurance (NHI) beneficiaries aged 40 to 79 years who lived in the catchment area of Ohsaki Public Health Center (Miyagi Prefecture) between October and December 1994. Japan has a universal healthcare system, and the NHI system is operated by local governments, enrolls individuals (eg, farmers, the self-employed, the retired, part-time workers, and their families) not covered by Employees’ Health Insurance, and pays for almost all medical services and medications.

This survey used a self-administered questionnaire on various health-related lifestyle factors. Among 54 996 eligible beneficiaries, 52 029 (95%) responded. We prospectively collected claims data (directly from the Miyagi NHI Organizations) on medical expenditures (inpatient and outpatient), insurance status, and survival status for all participants in the cohort during the study period of January 1995 through December 2007. This study was approved by the Ethics Committee of Kyoto University Graduate School of Medicine (Number E1059).

### Study population

Among the 52 029 participants in the baseline survey in 1994, we excluded 774 who died or withdrew from NHI before January 1, 1995. Of the 51 255 remaining participants, we selected all female lifelong nonsmokers (*n* = 17 803), who were identified by the question, “Are you a current smoker, a former smoker, or have never smoked?” All participants who selected the third option were classified as lifelong nonsmokers. We excluded women who provided incomplete information on ETS exposure at home (*n* = 634); those who made a proxy complete the baseline questionnaire (to avoid incorrect assessments made by a proxy; *n* = 2081); those exposed to ETS at work 3 or more days per week (*n* = 4538); those who were divorced or widowed (to avoid underestimating past ETS exposure at home; *n* = 1926); those who had left their job (to avoid underestimating past ETS exposure at work; *n* = 3284); those who died or withdrew in 1995, ie, the first follow-up year (to avoid extremely high or low monthly expenditures; *n* = 215); and those with a history of cancer, myocardial infarction, or stroke (*n* = 255). A total of 4870 women remained for analysis.

### Assessment of ETS exposure

We conducted a baseline questionnaire survey of all participants. The item on ETS exposure at home was worded, “How often are you exposed to environmental tobacco smoke at home?” Participants were asked to choose 1 of 5 options: almost every day, 3 to 4 days per week, 1 to 2 days per week, less than once per week, and rarely. For our analyses, we defined household high-level exposure to ETS as the first and second options, moderate-level exposure as the third and fourth options, and no exposure as the fifth option.

### Data analysis

With regard to baseline characteristics, differences between the 3 groups were examined using 1-way analysis of variance for duration of follow-up and the chi-square test for the other variables. The Fisher exact test was used when expected cell counts were less than 5.

The primary outcome was total (ie, inpatient plus outpatient care) average monthly medical expenditure per capita, because total expenditures attributable to ETS exposure can be considered to impose an economic burden on society. Total average monthly expenditure for each woman was calculated by dividing the accumulated expenditures through follow-up by number of months observed. A similar approach has been used in the econometric literature to assess medical expenditures related to smoking.^9^ In addition to total expenditure, we calculated and compared medical expenditures for inpatient and outpatient care. Outpatient expenditure included costs for drugs dispensed at pharmacies.

ETS exposure can cause fatal diseases, which incur considerable medical expenditures in the terminal phase.^24^ Calculating average monthly expenditure incurred by a mixed population of survivors and nonsurvivors creates a bias against the group with the higher mortality risk. Hence, we performed separate analyses for survivors and nonsurvivors.

Cost data are typically skewed to the right. Therefore, generalized linear models with a gamma distribution and log-link function were used to examine the relationship between medical expenditures and ETS exposure, after controlling for other variables.^25^ Gamma regression models have been shown to be multiplicative. Exponentiated coefficients were interpreted as cost ratio relative to the referent group. Gamma regression models removed zero-valued outcomes in the statistical software. Because the proportion of women with zero data for total and outpatient expenditures was small (6, 3, and 2 unexposed, moderately exposed, and highly exposed women, respectively), we added ¥1 to those who had zero expenditures to simplify the analyses. In contrast, inpatient expenditure data contained many zero-valued observations. Hence, we used logistic models to estimate the odds of any inpatient service use and then analyzed inpatient expenditures incurred only by women who had 1 or more hospitalizations.

Crude medical expenditures for moderately exposed and highly exposed women were compared with expenditures for unexposed women, which was used as the referent group. Then, analyses were age-adjusted (age strata: 40–49, 50–59, 60–69, and 70–79 years at baseline survey in 1994) and fully adjusted. The following variables were entered into the fully adjusted model: age stratum, body mass index (weight in kilograms divided by height in meters squared; <18.5, 18.5–24.9, ≥25.0),^11^^,^^26^ alcohol drinking status (never, former, currently drinking <3 *go*—a traditional Japanese unit of measure equal to 180 mL of Japanese sake and containing 22.8 g of ethanol—per week, currently drinking ≥3 *go* per week), tertiles of daily dietary energy intake (≤1163, 1163–1440, >1440 kcal/day), time spent walking per day (<30, 30–60, ≥60 minutes),^27^ self-rated health (good, mediocre, poor), marital status (married, unmarried), current job status (employed, unemployed), and education (≤9, >9 years). Time spent walking represented physical activity level.

The final multivariable model was similar to the fully adjusted model but included the interaction between exposure level and age stratum. This model with interaction enabled us to assess different ETS-attributable economic consequences by age stratum. Whether there were differences due to ETS exposure level was determined by cost ratios for each age stratum. The following SAS statements were used to calculate cost ratios of medical expenditures in the model with the interaction.


proc genmod data = ohsaki;

class ets age bmi alcohol diet walking healthrating marriage job education;
model expenditure = ets age bmi alcohol diet walking healthrating marriage job education ets*age / dist=gamma link=log type3;
run;


Median total monthly expenditures per capita and interquartile ranges are presented for each age stratum. All observed expenditures were not inflation-adjusted or discounted, because medical fees were revised slightly (−3.16% to 0.80%) every 2 years during the study period. A 2-sided test was used, and a *P*-value <0.05 was considered statistically significant. All expenditures are expressed as Japanese yen (¥107 = US$1 according to the purchasing power parity rate from the Organisation for Economic Co-operation and Development National Accounts database in 2011). All statistical analyses were performed using IBM SPSS version 18 (SPSS Inc., Chicago, IL, USA) and SAS version 9.2 (SAS Institute Inc., Cary, NC, USA).

## RESULTS

### Participant characteristics

Table 1 shows the baseline characteristics of women not exposed, moderately exposed, and highly exposed to ETS by survival status. The age stratum 60 to 69 years had the most women (*n* = 1664), and the age stratum 70 to 79 years had the fewest women (*n* = 507). Among all women (*n* = 4870), 1836 (38%) were not exposed to ETS at home, 1448 (30%) were moderately exposed, and 1586 (33%) were highly exposed. Among survivors, the largest groups in the age strata 40 to 49, 50 to 59, 60 to 69, and 70 to 79 years were women who were highly exposed, moderately exposed, unexposed, and unexposed, respectively.

**Table 1.** Characteristics of female nonsmokers

	Survivors				Nonsurvivors			
	
	Unexposed	Moderately exposed	Highly exposed	*P*-value^a^	Unexposed	Moderately exposed	Highly exposed	*P*-value^a^
No. of women	1687	1371	1494		149	77	92	
Age^b^ (years)								
40–49	369 (22)	361 (26)	506 (34)	<0.001	5 (3)	9 (12)	8 (9)	0.001
50–59	456 (27)	490 (36)	442 (30)		15 (10)	19 (25)	19 (21)	
60–69	641 (38)	426 (31)	454 (30)		72 (48)	25 (32)	46 (50)	
70–79	221 (13)	94 (7)	92 (6)		57 (38)	24 (31)	19 (21)	
Mean follow-up, months (SD)	138 (40)	136 (41)	132 (45)	0.001	92 (45)	99 (37)	90 (42)	0.33
Body mass index								
<18.5 kg/m^2^	63 (4)	39 (3)	43 (3)	0.14	6 (4)	4 (5)	5 (5)	0.29
18.5–24.9 kg/m^2^	1148 (68)	931 (68)	977 (65)		102 (68)	52 (68)	51 (55)	
≥25.0 kg/m^2^	476 (28)	401 (29)	474 (32)		41 (28)	21 (27)	36 (39)	
Alcohol drinking status								
Never	1355 (80)	1039 (76)	1151 (77)	0.06	127 (85)	61 (79)	79 (86)	0.09
Former	39 (2)	31 (2)	32 (2)		8 (5)	4 (5)	0 (0)	
Current, <3 *go* per week^c^	249 (15)	263 (19)	272 (18)		13 (9)	11 (14)	10 (11)	
Current, ≥3 *go* per week^c^	44 (3)	38 (3)	39 (3)		1 (1)	1 (1)	3 (3)	
Tertiles of dietary energy intake per day								
≤1163 kcal	592 (35)	443 (32)	452 (30)	0.07	73 (49)	28 (36)	34 (37)	0.19
1163–1440 kcal	551 (33)	461 (34)	521 (35)		40 (27)	23 (30)	25 (27)	
>1440 kcal	544 (32)	467 (34)	521 (35)		36 (24)	26 (34)	33 (36)	
Time spent walking per day								
<30 minutes	437 (26)	380 (28)	343 (23)	0.001	46 (31)	32 (42)	31 (34)	0.51
30–60 minutes	576 (34)	440 (32)	459 (31)		53 (36)	24 (31)	28 (30)	
≥60 minutes	674 (40)	551 (40)	692 (46)		50 (34)	21 (27)	33 (36)	
Self-rated health								
Good	1129 (67)	937 (68)	1019 (68)	0.60	71 (48)	36 (47)	47 (51)	0.91
Mediocre	265 (16)	210 (15)	245 (16)		25 (17)	16 (21)	16 (17)	
Poor	293 (17)	224 (16)	230 (15)		53 (36)	25 (32)	29 (32)	
Marital status								
Married	1614 (96)	1328 (97)	1472 (99)	<0.001	143 (96)	76 (99)	89 (97)	0.64
Unmarried	73 (4)	43 (3)	22 (1)		6 (4)	1 (1)	3 (3)	
Current job status								
Employed	916 (54)	784 (57)	788 (53)	0.05	57 (38)	29 (38)	40 (43)	0.67
Unemployed	771 (46)	587 (43)	706 (47)		92 (62)	48 (62)	52 (57)	
Education								
≤9 years	819 (49)	658 (48)	746 (50)	0.56	91 (61)	41 (53)	58 (63)	0.39
>9 years	868 (51)	713 (52)	748 (50)		58 (39)	36 (47)	34 (37)	

Exposure indicates exposure to environmental tobacco smoke in homes.

Values are expressed as number (column percentage), unless otherwise indicated.

Because of rounding, percentages may not add up to 100%.

^a^Differences among the 3 groups (analysis of variance for mean follow-up months and chi-square test for categorical variables).

^b^Age at baseline survey in 1994.

^c^1 *go* is equal to 180 mL of Japanese sake and contains 22.8 g of ethanol.

A total of 318 (7%) women died during the follow-up period; 22 (2%) died among 1258 women aged 40 to 49, and 100 (20%) died among 507 women aged 70 to 79. Among survivors, mean follow-up was 135 months; among nonsurvivors, mean follow-up was 93 months. Among survivors, there were significant differences in time spent walking and the proportion of married women among the 3 groups.

### Medical expenditures for survivors

Median total monthly medical expenditures per capita ranged from ¥5900 (age stratum 40–49) to ¥29 900 (age stratum 70–79) among survivors (Table 2).

**Table 2.** Monthly medical expenditures for survivors and cost ratios of expenditures for those exposed to ETS at home relative to unexposed survivors

	Median total expenditure (IQR), JPY	Total expenditure		Hospitalization		Inpatient expenditure^a^		Outpatient expenditure	
			
		Cost ratio (95% CI)	*P*-value	Odds ratio (95% CI)	*P*-value	Cost ratio (95% CI)	*P*-value	Cost ratio (95% CI)	*P*-value
Ratio across all age strata									
Crude ratio									
Moderately exposed		0.90 (0.83–0.97)	0.006	0.86 (0.75–0.99)	0.04	0.84 (0.74–0.96)	0.009	0.94 (0.88–1.01)	0.11
Highly exposed		0.92 (0.85–0.99)	0.02	0.83 (0.72–0.95)	0.009	1.10 (0.97–1.25)	0.14	0.89 (0.83–0.96)	0.002
Age-adjusted ratio^b^									
Moderately exposed		1.01 (0.94–1.08)	0.89	0.97 (0.83–1.12)	0.65	0.91 (0.81–1.04)	0.17	1.04 (0.97–1.11)	0.30
Highly exposed		1.05 (0.98–1.13)	0.17	0.96 (0.83–1.10)	0.53	1.15 (1.02–1.31)	0.02	1.03 (0.96–1.10)	0.38
Fully adjusted ratio^c^									
Moderately exposed		0.98 (0.91–1.05)	0.52	0.96 (0.83–1.12)	0.63	0.93 (0.82–1.06)	0.26	1.00 (0.94–1.07)	0.90
Highly exposed		1.01 (0.94–1.08)	0.87	0.94 (0.81–1.09)	0.43	1.18 (1.05–1.34)	0.007	0.98 (0.92–1.04)	0.50
Ratio in each age stratum^d^									
Age stratum 40–49 years	5900 (2800–13 100)								
Moderately exposed		0.91 (0.79–1.05)	0.20	0.89 (0.65–1.21)	0.46	0.84 (0.63–1.11)	0.21	0.97 (0.85–1.11)	0.65
Highly exposed		0.99 (0.87–1.12)	0.83	0.73 (0.54–0.98)	0.03	1.22 (0.93–1.59)	0.15	1.00 (0.89–1.13)	0.97
Age stratum 50–59 years	11 600 (4800–21 300)								
Moderately exposed		1.07 (0.94–1.21)	0.29	1.18 (0.91–1.55)	0.22	0.99 (0.78–1.26)	0.95	1.07 (0.95–1.20)	0.27
Highly exposed		0.99 (0.87–1.13)	0.91	1.07 (0.81–1.42)	0.63	1.04 (0.81–1.33)	0.77	0.98 (0.87–1.10)	0.68
Age stratum 60–69 years	21 900 (11 800–34 900)								
Moderately exposed		0.93 (0.82–1.04)	0.21	0.81 (0.63–1.03)	0.09	0.96 (0.79–1.17)	0.68	0.95 (0.85–1.06)	0.37
Highly exposed		0.94 (0.84–1.06)	0.33	0.99 (0.77–1.26)	0.92	1.06 (0.88–1.28)	0.54	0.92 (0.82–1.02)	0.12
Age stratum 70–79 years	29 900 (16 800–45 300)								
Moderately exposed		0.98 (0.78–1.24)	0.90	1.11 (0.67–1.85)	0.69	0.74 (0.53–1.03)	0.08	1.08 (0.87–1.34)	0.51
Highly exposed		1.43 (1.13–1.81)	0.003	1.18 (0.71–1.98)	0.53	1.94 (1.38–2.74)	<0.001	1.17 (0.94–1.46)	0.17

All ratios are expressed as ratio relative to women unexposed to ETS. Age indicates age at baseline survey in 1994.

ETS, environmental tobacco smoke; IQR, interquartile range; JPY, Japanese yen.

^a^Inpatient expenditures are compared among women who had 1 or more hospitalizations.

^b^Adjusted for 10-year age stratum.

^c^Adjusted for age, body mass index, alcohol drinking status, dietary energy intake, time spent walking, self-rated health, marital status, current job status, and education.

^d^Computed from a model that includes interaction term (ETS exposure level × 10-year age stratum) in addition to ETS exposure level, age, body mass index, alcohol drinking status, dietary energy intake, time spent walking, self-rated health, marital status, current job status, and education.

Crude, age-adjusted, and fully adjusted (the model without the interaction) cost ratios for moderately exposed and highly exposed survivors, as compared with unexposed survivors, are shown in Table 2. Cost ratios of total (ie, inpatient plus outpatient care), inpatient and outpatient expenditures, and odds ratios of hospitalization were calculated for moderately exposed and highly exposed women in relation to unexposed women, which was used as the referent category.

We found significant differences in some crude total, inpatient, and outpatient expenditures associated with ETS exposure. For example, crude total expenditures were lower for moderately exposed and highly exposed women than for unexposed women. In contrast, we found no significant difference in age-adjusted or fully adjusted total expenditures, regardless of exposure, although age-adjusted and fully adjusted inpatient expenditures were higher for highly exposed women than for unexposed women.

There were significant differences in the fully adjusted model with the interaction term between exposure level and age stratum (Table 2). Total expenditure for age stratum 70 to 79 years was higher for highly exposed women than for unexposed women after adjusting for other variables (cost ratio, 1.43; 95% CI, 1.13–1.81). The difference in total expenditure between unexposed and moderately exposed women was not significant.

When inpatient and outpatient care were analyzed separately, inpatient expenditure was higher for highly exposed women than for unexposed women (cost ratio, 1.94; 95% CI, 1.38–2.74), although there was no significant association between probability of hospitalization and exposure level. We found no difference in adjusted outpatient expenditures for age stratum 70 to 79 years, regardless of exposure.

In the age strata 40 to 49, 50 to 59, and 60 to 69 years, total, inpatient, and outpatient medical expenditures did not significantly differ, regardless of exposure (Table 2). However, inpatient expenditures tended to be higher for highly exposed women than for unexposed women among all these strata.

### Medical expenditures for nonsurvivors

Median total monthly medical expenditures per capita ranged from ¥48 200 (age stratum 40–49) to ¥69 500 (age stratum 60–69) among nonsurvivors (Table 3).

**Table 3.** Monthly medical expenditures for nonsurvivors and cost ratios of expenditures for those exposed to ETS at home relative to unexposed nonsurvivors

	Median total expenditure (IQR), JPY	Total expenditure		Hospitalization		Inpatient expenditure^a^		Outpatient expenditure	
			
		Cost ratio (95% CI)	*P*-value	Odds ratio (95% CI)	*P*-value	Cost ratio (95% CI)	*P*-value	Cost ratio (95% CI)	*P*-value
Ratio across all age strata									
Crude ratio									
Moderately exposed		0.68 (0.53–0.87)	0.002	0.88 (0.33–2.32)	0.79	0.71 (0.52–0.96)	0.02	0.65 (0.49–0.85)	0.002
Highly exposed		0.85 (0.68–1.08)	0.18	0.49 (0.22–1.11)	0.09	0.91 (0.68–1.22)	0.52	0.88 (0.68–1.14)	0.33
Age-adjusted ratio^b^									
Moderately exposed		0.69 (0.54–0.88)	0.003	0.94 (0.35–2.54)	0.90	0.70 (0.51–0.95)	0.02	0.70 (0.53–0.92)	0.01
Highly exposed		0.84 (0.66–1.06)	0.14	0.51 (0.22–1.18)	0.12	0.89 (0.66–1.20)	0.45	0.85 (0.66–1.10)	0.22
Fully adjusted ratio^c^									
Moderately exposed		0.69 (0.54–0.87)	0.002	1.11 (0.39–3.19)	0.85	0.66 (0.48–0.90)	0.009	0.76 (0.59–0.99)	0.04
Highly exposed		0.90 (0.71–1.13)	0.36	0.57 (0.23–1.39)	0.22	0.92 (0.67–1.26)	0.60	0.93 (0.73–1.18)	0.54
Ratio in each age stratum^d^									
Age stratum 40–49 years	48 200 (16 800–72 200)								
Moderately exposed		0.73 (0.28–1.86)	0.50	NA		0.52 (0.16–1.66)	0.27	1.14 (0.42–3.09)	0.79
Highly exposed		0.97 (0.37–2.49)	0.94	NA		1.30 (0.40–4.17)	0.66	0.57 (0.21–1.55)	0.27
Age stratum 50–59 years	57 600 (22 500–111 400)								
Moderately exposed		0.58 (0.32–1.05)	0.07	1.60 (0.18–13.89)	0.67	0.70 (0.33–1.48)	0.35	0.41 (0.22–0.77)	0.005
Highly exposed		1.05 (0.58–1.92)	0.87	0.51 (0.07–3.64)	0.50	1.54 (0.70–3.38)	0.28	0.60 (0.32–1.12)	0.11
Age stratum 60–69 years	69 500 (34 600–114 100)								
Moderately exposed		0.75 (0.51–1.11)	0.15	NA		0.78 (0.48–1.26)	0.31	0.65 (0.43–0.98)	0.04
Highly exposed		0.78 (0.57–1.07)	0.12	0.61 (0.19–1.99)	0.41	0.75 (0.49–1.14)	0.17	0.91 (0.65–1.27)	0.57
Age stratum 70–79 years	52 700 (32 400–108 000)								
Moderately exposed		0.69 (0.46–1.05)	0.08	0.41 (0.08–2.18)	0.29	0.60 (0.34–1.05)	0.07	1.03 (0.67–1.59)	0.90
Highly exposed		1.03 (0.65–1.64)	0.90	0.71 (0.09–5.50)	0.74	0.87 (0.48–1.58)	0.65	1.35 (0.83–2.18)	0.23

All ratios are expressed as ratio relative to women unexposed to ETS. Age indicates age at baseline survey in 1994.

ETS, environmental tobacco smoke; IQR, interquartile range; JPY, Japanese yen; NA, not applicable.

^a^Inpatient expenditures are compared among women who had 1 or more hospitalizations.

^b^Adjusted for 10-year age stratum.

^c^Adjusted for age, body mass index, alcohol drinking status, dietary energy intake, time spent walking, self-rated health, marital status, current job status, and education.

^d^Computed from a model that includes interaction term (ETS exposure level × 10-year age stratum) in addition to ETS exposure level, age, body mass index, alcohol drinking status, dietary energy intake, time spent walking, self-rated health, marital status, current job status, and education.

Crude, age-adjusted, and fully adjusted (the model without the interaction) cost ratios for moderately exposed and highly exposed nonsurvivors, as compared with unexposed nonsurvivors, are shown in Table 3. Crude, age-adjusted, and fully adjusted total expenditures were lower for moderately exposed nonsurvivors than for unexposed nonsurvivors.

Table 3 also shows cost ratios of medical expenditures for nonsurvivors in the fully adjusted model with the interaction between exposure level and age stratum. In all age strata, including age stratum 70 to 79 years, we found no significant difference in total expenditure regardless of exposure, although outpatient expenditures for the age strata 50 to 59 and 60 to 69 years were lower for moderately exposed nonsurvivors than for unexposed nonsurvivors.

## DISCUSSION

To calculate expenditures attributable to ETS, we analyzed medical expenditures incurred by female nonsmokers who were not exposed, moderately exposed, and highly exposed to ETS in the home. Among highly exposed survivors aged 70 to 79 years, we found that a substantial increase in total medical expenditure was possibly attributable to ETS exposure at home. This excess expenditure suggests a significant age-specific association between ETS exposure and total medical expenditure.

The association of ETS exposure with economic impact is consistent with known clinical relationships and provides an explanation for the economic burden. Although our significant findings regarding total expenditures are limited to older survivors, they support the results of previous simulation studies^16^^–^^21^ that estimated the economic burden caused by ETS-attributable diseases at the national, regional, and state levels rather than the individual level.

### Interpretation of findings

High ETS exposure resulted in significantly higher inpatient but not outpatient expenditures among survivors aged 70 to 79 years, which suggests that the excess total expenditure arose from treatment for serious but nonfatal diseases rather than from treatment for relatively minor disorders.

ETS exposure significantly increased total medical expenditures only among survivors aged 70 to 79 years. One plausible explanation for this result is that it takes many years to produce a significant difference in total medical expenditures between unexposed and highly exposed adults, because the harm of ETS is subtle in comparison to active smoking. Another likely explanation is that diseases result in higher morbidity among older versus younger adults. Notably, among women aged 40 to 69 years, highly exposed women tended to incur higher inpatient expenditures than unexposed women, which suggests that serious but nonfatal diseases attributable to ETS are already present among highly exposed women in these age strata.

In contrast to survivors, we found no significant age-specific association between total medical expenditures incurred by nonsurvivors and ETS exposure, perhaps because ETS exposure does not increase costs of therapy for fatal diseases, although exposure increases the incidence of such diseases. In contrast to our findings among nonsurvivors, ETS-attributable excess total expenditures among survivors may arise from the accumulation of excess morbidities from nonfatal and near-fatal diseases.

The results for nonsurvivors in age strata 50 to 59 and 60 to 69 years showed that moderately exposed nonsurvivors incurred lower outpatient expenditures as compared with unexposed nonsurvivors, possibly because relatively low doses of toxins inhaled from ETS are pathophysiologically sufficient to elicit a strong acute effect on the cardiovascular system, whereas lung cancer is caused by long-term exposure.^28^ This evidence suggests that ETS exposure causes rapid onset of coronary heart disease and subsequent death in these age strata, which may account for the lower monthly outpatient expenditure.

### Strengths and limitations of the study

Our study has several advantages as compared with previous studies. To the best of our knowledge, this is the first study using directly observed long-term individual-level health insurance records to show a significant association between ETS exposure and medical expenditures among adults. Previous studies estimated population-level ETS-attributable expenditures by using economic models of mixed results from multiple databases, such as the published literature and macrodata on ETS exposure, increased morbidity, and medical costs.^16^^–^^21^ We showed the individual-level economic burden imposed by ETS exposure by means of comparison within a single cohort.

Furthermore, we followed a large cohort for a long period. The expenditures in our analyses were accurate because we obtained health insurance claims data directly from the Miyagi NHI Organizations, which included information on almost all available medical services. Long-term observation allowed average monthly expenditure to be unaffected by short-term incidental use of medical services.

Our study has several limitations. First, assessment of ETS exposure was based on a questionnaire survey. Misclassification of exposure status is a concern in studies that use only questionnaires. Quantitative information on ETS exposure is less reliable in questionnaires, but information on whether exposure is heavy or light is relatively reliable.^29^^,^^30^ Second, the questionnaire in our study focused on ETS exposure at 1 time point, as we assumed that exposure status at baseline was correlated with past exposure. To ensure the correctness of this assumption, we excluded women with a change in job status or marital status during their lifetime. Nevertheless, further research based on long-term continuous ETS exposure measurement is required. Third, all medical expenditures were included in our analysis. There were no available cost data for specific diseases such as lung cancer and coronary heart disease. However, diseases attributable to ETS exposure range from life-threatening diseases to relatively minor disorders (eg, respiratory tract symptoms).^31^ This is similar to active smoking, which has been reported to be relevant to many diseases, including major smoking-related diseases.^32^ Previous studies of the association between active smoking and medical expenditures have also addressed overall disease burden.^9^^,^^10^ Furthermore, if we analyzed expenditures for specific diseases that were strongly associated with ETS exposure, we might also find significant relationships between ETS exposure and total expenditures for younger women. Future studies of real-world medical expenditures for diseases strongly associated with ETS exposure may be needed to verify the results of previous studies that simulated the excess costs attributable to ETS by combining ETS-attributable diseases and associated medical costs. Finally, long-term care (LTC) insurance claims data were not available. Japan has a LTC insurance system that supports elderly adults living at home or in nursing-care facilities. We believe that women that incur more medical expenditures for treatment of diseases due to ETS exposure may also use more LTC services than women unexposed to ETS.

We examined household ETS exposure because previous cohort studies of the health effects of ETS focused on ETS exposure at home or at work.^1^^,^^2^^,^^5^ How applicable are our results to ETS exposure outside the home? Considerable evidence of increased mortality and morbidity related to ETS exposure has also been obtained from exposure in workplaces and public places.^4^^,^^5^^,^^33^ We believe that ETS exposure in settings other than the home also increases medical expenditures. Medical expenditures attributable to ETS exposure in the workplace and other settings need to be explored.

This study analyzed the self-employed, part-time workers, the unemployed, and their families. These groups may spend more time at home than corporate employees, who are covered by Employees’ Health Insurance. Thus, the effects of household ETS might be more severe in the present study population than in corporate employees and their families.

### Conclusions

We found that severe household ETS exposure results in excess total medical expenditures. Surviving female nonsmokers aged 70 to 79 years who were highly exposed to ETS at home incurred significantly higher total medical expenditures than those living in smoke-free households. The present study provides information on the economic burden of ETS, although significant findings regarding total expenditure are limited to surviving older women. This information should help policymakers to develop strategies that reduce secondhand smoke and hasten the economically attractive goal of eliminating ETS. Further research is required to examine the association between accumulated ETS exposure and medical expenditure.

## ONLINE ONLY MATERIALS

The Japanese-language abstract for articles can be accessed by clicking on the tab labeled Supplementary materials at the journal website http://dx.doi.org/10.2188/jea.JE20120072.

Abstract in Japanese.
