# Supplementary material for: Burden of Household Environmental Tobacco Smoke on Medical Expenditure for Japanese Women: A Population-Based Cohort Study
Source: J Epidemiol. 2013 Jan 5;23(1):55–62. doi: 10.2188/jea.JE20120072 (PMC3700236; doi:10.2188/jea.JE20120072)
Supplement: Abstract in Japanese. [file je-23-055-s001.pdf]

家庭内の受動喫煙は医療費を増やす：地域住民を追跡したコホート研究

森島敏隆[1]、今中雄一[1]、大坪徹也[1]、林田賢史[2]、渡邉崇[3]、辻一郎[3]

[1] 京都大学大学院医学研究科医療経済学分野、[2] 産業医科大学病院 医療情報部、[3] 東北大学大学院医学系研究科社会医学講座公衆衛生学分野

【背景】受動喫煙は数多くの喫煙関連疾患の罹患リスクを上昇させることが知られている。受動喫煙と医療費の関係を検証した先行研究はいずれも計量モデルによって推計したものであり、実証研究は国内外において報告されていない。本研究の目的は、能動喫煙をしたことのない成人女性を対象に、家庭での受動喫煙が医療費を増やすかどうかを明らかにすることである。

【方法】宮城県大崎保健所管内に居住する国民健康保険加入者を追跡した大崎コホート国保研究のデータを用いた。1994 年に 40〜79 歳の加入者に受動喫煙に関する情報を質問紙で調査した。加入者の医療費データを保険者を通して 1995 年〜2007 年に収集した。一般化線形モデルを用いて様々な交絡因子を調整した上で、生存者/非生存者別に受動喫煙の高度暴露群、中等度暴露群、非暴露群の医療費を解析した。

【結果】4870 人の成人女性の医療費を分析した。70〜79 歳の女性のうち、高度暴露群の生存者の月間総医療費（入院医療費＋入院外医療費＋調剤医療費）は非暴露群の生存者の医療費に比べて有意に高かった。中等度暴露群と非暴露群の間に有意差はなかった。他の年齢階級の生存者と全年齢階級の非生存者の医療費で受動喫煙の暴露レベルによる有意差を認めなかった。

【結論】受動喫煙によって医療費が増加することを個人レベルの観察データに基づいて実証した。受動喫煙によってもたらされる経済的負担を示した本研究は、受動喫煙を防止する政策を形成するための１つの客観的根拠となるだろう。

キーワード：受動喫煙、タバコ煙公害、医療費、診療報酬明細書、保健医療政策、公衆衛生
